# The Role of MicroRNAs in Ovarian Cancer

**DOI:** 10.1155/2014/249393

**Published:** 2014-09-10

**Authors:** Yasuto Kinose, Kenjiro Sawada, Koji Nakamura, Tadashi Kimura

**Affiliations:** Department of Obstetrics and Gynecology, Osaka University Graduate School of Medicine, Osaka 5650871, Japan

## Abstract

Ovarian cancer is the most lethal of malignant gynecological tumors. Its lethality may be due to difficulties in detecting it at an early stage and lack of effective treatments for patients with an advanced or recurrent status. Therefore, there is a strong need for prognostic and predictive markers to diagnose it early and to help optimize and personalize treatment. MicroRNAs are noncoding RNAs that regulate target genes posttranscriptionally. They are involved in carcinogenesis, cell cycle, apoptosis, proliferation, invasion, metastasis, and chemoresistance. The dysregulation of microRNAs is involved in the initiation and progression of human cancers including ovarian cancer, and strong evidence that microRNAs can act as oncogenes or tumor suppressor genes has emerged. Several microRNA signatures that are unique to ovarian cancer have been proposed, and serum-circulating microRNAs have the potential to be useful diagnostic and prognostic biomarkers. Various microRNAs such as those in the miR-200 family, the miR-199/214 cluster, or the let-7 paralogs have potential as therapeutic targets for disseminated or chemoresistant ovarian tumors. Although many obstacles need to be overcome, microRNA therapy could be a powerful tool for ovarian cancer prevention and treatment. In this review, we discuss the emerging roles of microRNAs in various aspects of ovarian cancer.

## 1. Introduction

Ovarian cancer is the most lethal gynecological malignancy in developed countries. In the United States, ovarian cancer is the fifth leading cause of cancer death in women, with estimated 14270 deaths in 2014 [1]. It is often diagnosed at a late stage with peritoneal dissemination and massive ascites, and despite combined treatments of aggressive cytoreductive surgery with platinum- and taxane-based chemotherapy the 5-year survival rate remains only 30% [1]. One reason for this high mortality rate is the lack of an early detection method for ovarian cancer. Indeed, the 5-year survival rate at stages I-II is estimated to be approximately 90%. While pelvic examination, transvaginal ultrasonography, and serum CA125 are performed during routine diagnostic procedures, they have failed to detect the disease at an early stage and thus reduce the mortality [2]. Therefore, new approaches for detecting early stage ovarian cancer are urgently needed. Another cause of the high mortality rate is the difficulty of treating disseminated or recurrent ovarian cancer. Although the clinical response rate after platinum- and taxane-based chemotherapy is usually high initially, subsequent relapses and repetitive treatments using cytotoxic chemotherapies lead to an acquired resistance to those treatments. Therefore, most patients that suffer relapses finally succumb to their disease [3]. At the molecular level, a number of interesting genes and pathways that may play essential roles in the pathogenesis of ovarian cancer have already been identified. Many could serve as molecular targets for therapies, although effective treatments that substantially extend overall patient survival have not been established so far. Among these, the recently discovered microRNAs (miRNAs or miRs) constitute a novel layer of gene expression regulation and have been implicated in the etiology of ovarian cancer. This review summarizes the ways in which microRNAs are involved in the pathogenesis of ovarian cancer and discusses cumulative efforts to apply them to the creation of novel diagnostic tools or promising future therapies.

## 2. MicroRNAs and Cancer

MicroRNAs (miRs) are approximately 22-nucleotide noncoding RNAs, are highly conserved among a wide range of species, and are generally involved in posttranscriptional gene regulation. MiRs negatively regulate genes expression by binding to the 3′-untranslated region (UTR) of target mRNAs. Since miRs do not require perfectly complementary target sites and recognize short sites complementary to their 5′-seed region (nucleotides 2–8 of the miRs), one miR can regulate hundreds of mRNAs and multiple miRs can regulate an individual mRNA [4]. MiRs are predicted to regulate approximately 60% of all human genes and are involved in processes such as development, differentiation, metabolism, proliferation, cell cycle, and inflammation and the immune system [5–8]. Currently, it is well known that miRs can be upregulated or downregulated in various human cancers. Overexpressed miRs may function as oncogenes by downregulating tumor suppressor genes, whereas the downregulated miRs may act as tumor suppressor genes by negatively regulating oncogenes [9]. Important insights into the functions of miRs in cancer have been provided through the demonstration that they are involved in known oncogenic pathways. Three human* RAS* oncogenes (*H-*,* K-*, and* N-RAS*) contain binding sites for the let-7 family of miRs in their 3′UTR [10]. Interestingly, the miRs of the let-7 family, which are typically downregulated in various tumors, have been shown to negatively regulate the* RAS* oncogenes, thereby acting as tumor suppressors [10, 11]. MiR-15 and miR-16 have been shown to target the* BCL2* oncogene, leading to its downregulation and, consequently, resulting in apoptosis in leukemic cells [12]. MiR-221 and miR-222 are examples of miRs that act as oncogenes. They do so by targeting and inhibiting the expression of the tumor suppressor gene,* p27Kip* [13]. High levels of these miRs were shown to result in low p27 protein expression and increased proliferation of cancer cells. There is also evidence of a role of miR in p53-induced cell death. It has been shown that p53 transcriptionally induces miR-34 expression, and this induction is important in p53-mediated apoptosis of cancer cells [7, 14, 15]. These studies only represent a fraction of the explosion of publications emphasizing the role of miRs in cancer biology and showing miR dysregulation in various malignancies, including ovarian cancer.

## 3. Drosha/Dicer and Ovarian Cancer

Drosha and Dicer are essential for the biogenesis of miRNA. Drosha, an RNase III enzyme, cleaves the pri-miRNA and releases a hairpin-structured pre-miRNA in the nucleus. After the pre-miRNA is exported to the cytoplasm, Dicer, another RNase III enzyme, cleaves the pre-miRNA and releases the miRNA duplexes. When mature miRNA duplexes are produced, they associate with Argonaute (Ago) proteins and form the RNA-induced silencing complex (RISC), resulting in the degradation or translational repression of specific target mRNAs. In 2008, Merritt et al. measured the mRNA levels of Drosha and Dicer in 111 clinical samples of epithelial ovarian cancer and analyzed the prognostic values [16]. Low Dicer expression is significantly associated with advanced stage ovarian cancer and low Drosha expression with suboptimal surgery. Low Dicer expression is an independent predictor of disease-specific survival in multivariate analysis, as well as high-grade histological finding and chemotherapy resistance. These results suggested that impaired processing of miRs by Dicer and Drosha is involved in the tumorigenesis of ovarian cancer and leads to poor clinical outcomes. Vaksman et al. showed that the metastatic sites of differential expression of Drosha, Dicer, Ago1, and Ago2 in ovarian cancers are different from those in primary carcinomas [17]. In their study, higher Ago2 protein expression in ovarian cancer before chemotherapy correlated with shorter progression free survival. The study group saw similar trends for both Ago1 and Ago2 with respect to overall survival, suggesting a pivotal role of these molecules in ovarian cancer progression. Kim et al. proved that high-grade serous carcinomas could arise from the Fallopian tube in mice by conditionally knocking out Dicer and phosphatase and tensin homolog (*Pten*), which is a key negative regulator of the PI3K pathway [18]. Collectively, these reports demonstrate that enzymes and proteins involved in miR biogenesis and processing are closely related to development and progression in ovarian cancer.

## 4. miR Expression Profiles in Ovarian Cancer

In 2008, studies using miR microarrays, cDNA microarrays, and tissue arrays demonstrated genome-wide transcriptional changes in ovarian cancer [19]. Numerous miRs are markedly downregulated in advanced stages or high-grade ovarian cancer, suggesting that miRs are involved in malignant transformation and tumor progression. Both genomic copy number loss and epigenetic alteration may account for this downregulation and contribute to genome-wide transcriptional dysregulation. The authors compared the miR expression profiles of 18 epithelial ovarian cancer (EOC) cell lines and 4 immortalized ovarian surface epithelium (IOSE) primary cultures. They showed that the expression levels of 35 miRs were significantly different between the EOC lines and IOSE lines. Of these, 31 miRs (88.6%, 31/35) were downregulated in the EOC lines compared with the IOSE lines, including the tumor suppressor miRs, let-7d [10], and miR-127 [20].

Iorio et al. reported different miR expression profiles between ovarian cancer tissues/cell lines and normal tissues. Of 29 miRs, they showed that only 4 (miR-141, miR-200a, miR-200b, and miR-200c) were upregulated and 25 were downregulated, including miR-199a, miR-140, miR-145, and miR-125b-1 in the cancer samples [21]. They also found that miR signatures were different between ovarian carcinoma histotypes (serous, endometrioid, clear cell, and mucinous). Calura et al. analyzed miR profiles characteristic of each EOC histotype at stage 1 and found robust miR markers for clear cell and mucinous histotypes. The clear cell histotype is characterized by higher expression of miR-30a-5p and miR-30a-3p, whereas mucinous histotype displays higher levels of miR-192 and miR-194 [22]. Nam et al. reported the miR expression profiles of 20 serous ovarian carcinomas using a miRNA microarray and compared them with normal samples [23]. In ovarian cancer, 11 miRs were upregulated (miR-16, miR-20a, miR-21, miR-23a, miR-23b, miR-27a, miR-93, miR-141, miR-200a, miR-200b, and miR-200c) and 12 were downregulated (miR-10b, miR-26a, miR-29a, miR-99a, miR-100, miR-125a, miR-125b, miR-143, miR-145, miR-199a, miR-214, and let-7b). Thus, these reports identified similar sets of dysregulated miRs. Vang et al. analyzed the miR expression profiles of primary serous ovarian cancers and their respective omental metastases using miRNA qPCR arrays [24]. Seventeen miRs showed differential expression in omental lesions compared to primary tumors. Among these, miR-146a and miR-150 were significantly increased in omental metastases, regulating enhancement of spheroid formation and cisplatin resistance.

The Cancer Genome Atlas project has analyzed mRNA expression, miRNA expression, promoter methylation, and DNA copy number in a total of 489 high-grade serous ovarian adenocarcinomas [25]. They reported that high-grade serous ovarian cancer was characterized by* TP53* mutations in almost every tumor (96%). In addition, there was a low but statistically significant prevalence of recurrent somatic mutations in 8 other genes including* NF1*,* BRCA1*,* BRCA2*,* RB1*, and* CDK12*. They also showed that ovarian cancers could be separated into 4 transcriptional subtypes, 3 miR subtypes, and 4 promoter methylation subtypes. Integrated genomic analysis revealed a miRNA-regulatory network that defined a robust integrated mesenchymal subtype associated with poor survival in 459 cases of serous ovarian cancer and 560 cases independent of cohort data [26]. Eight key miRs (miR-25, miR-29c, miR-101, miR-128, miR-141, miR-182, miR-200a, and miR-506) were identified and predicted to regulate 89% of the targets in this network. Recently, Davidson et al. summarized the clinical and diagnostic roles of miRs in ovarian carcinoma in their review of approximately 100 publications [27]. In addition, various miRs have also been identified as potential prognostic indicators and promise utility in future practice. These are summarized in Table 1 [28–40].

## 5. Plasma/Serum miRs as Early Diagnostic Biomarkers for Ovarian Cancer

Ovarian cancer is a disease for which noninvasive serum screening tests are highly desirable for early stage cancer detection. Emerging evidence shows that miRs exist not only in cells but also in circulating blood, reflecting tissue or organ conditions. miRs generated in the cytoplasm can not only affect the function of the cell in which they are produced, but they can also be released into the blood stream and are taken up to regulate the gene expression of distant target cells [41]. Circulatory miRs in blood are resistant to the degradation of RNase enzyme and remain stable [42, 43]. Lawrie et al. first described serum miRs in cancer patients and suggested that miRs have potential as minimally invasive diagnostic markers for diffuse large B cell lymphoma (DLBCL) and possibly other cancers [44]. They found that the levels of miR-21, miR-155, and miR-210 in serum from DLBCL patients were higher than in healthy controls. In addition, high miR-21 expression was associated with relapse-free survival.

The potential of circulating miRs as cancer biomarkers depends on their high stability and their capacity to reflect tumor status and predict therapy response [43]. Many studies have determined that circulating miRs remain stable after being subjected to harsh conditions that would normally degrade RNAs, such as boiling, extreme pH levels, extended storage time, and repetitive freeze-thaw cycles. This incredible stability is partly explained by the association of the miRs with protein complexes such as Ago2 and the presence of these small RNAs in circulating microvesicles such as exosomes. Arroyo et al. found that most circulating miRs in plasma are cofractionated with Ago2, suggesting that circulating Ago2 complexes are responsible for the stability of plasma miR [45]. The authors reported that approximately 90% of miRs in circulation are present in a non-membrane-bound form consistent with a RISC complex. Other proteins may also be associated with circulating miRs. Vickers et al. presented evidence that high-density lipoprotein (HDL) transports endogenous miRs and delivers them to recipient cells with functional targeting capabilities and that the cellular export of miRs to HDL is regulated by neutral sphingomyelinase [46]. While only 10% of circulating miRs are packaged in microparticles such as exosomes, recent research has revealed that exosomal miRs can affect many aspects of physiological and pathological conditions. Taylor and Gercel-Taylor reported that the miR signatures of tumor-derived exosomes have the potential to be used as diagnostic biomarkers of ovarian cancer [47]. Exosomes are small (30–100 nm) lipoprotein vesicles that exist in body fluids. They contain proteins, mRNAs, and miRs and are thought to play important roles in intercellular communication. The researchers compared the expression profiles of 8 miRs (miR-21, miR-141, miR-200a, miR-200b, miR-200c, miR-203, miR-205, and miR-214) between cancer tissues and exosomes collected from the peripheral sera of the corresponding patients, since these had been previously demonstrated to be overexpressed in ovarian cancer. They showed that exosomal miR profiles from ovarian cancer patients were elevated, whereas the exosomal miRs could not be detected in normal healthy controls.

Resnick et al. compared 21 serum miRs between epithelial ovarian cancer patients and healthy controls [48]. MiR-21, miR-29a, miR-92, miR-93, and miR-126 were significantly overexpressed in the serum of ovarian cancer patients compared to controls, while miR-99b, miR-127, and miR-155 were significantly underexpressed. Häusler et al. investigated the whole blood-derived miR profiles of ovarian cancer patients [49]. A comparison between ovarian cancer patients and healthy controls detected 147 significantly dysregulated miRs. In particular, miR-30c-1-3p was significantly upregulated and miR-181a-3p, miR-342-3p, and miR-450b-5p were significantly downregulated in ovarian cancer patients. Kan et al. found that miR-200a, miR-200b, and miR-200c were significantly elevated in the serum of patients and suggested that their presence could be used as a predictor of ovarian cancer [50]. Chung et al. reported that serum miR-26a, miR-132, miR-145, and let-7b could be considered potential candidates as novel biomarkers of serous ovarian cancer [51]. Suryawanshi et al. identified 3 distinct miR signatures between healthy controls, patients with endometriosis, and patients with endometriosis-associated ovarian cancer [52]. They suggested that these signatures might serve as useful diagnostic markers for the discrimination of these diseases, which is often clinically difficult. Zheng et al. showed that plasma miR-205 and let-7f could be used as biomarkers for ovarian cancer detection, especially in patients with stage 1 disease [53]. These efforts strongly support the idea that the detection of ovarian cancer-associated miRs from the peripheral blood could become a valuable method for early diagnosis of this disease in future clinical practice. Improving the sensitivity and lowering the cost of such detection methods are both key goals for advancing the application of detecting serum miR in cancer patients. Plasma/serum miRs that are potentially useful for the diagnosis or detection of ovarian cancer are summarized in Table 2 [47–53].

## 6. Therapeutic Potential of miRs That Inhibit Ovarian Cancer Progression

With the progress in cancer profiling, treatments will soon be customized for each individual. Because each miR regulates the expression of hundreds of different genes, miRs can function as master coordinators, efficiently regulating and coordinating multiple cellular pathways and processes [5]. Thus, miRs have been suggested as possible therapeutic armaments against cancer. The therapeutic application of miRs involves two strategies, inhibiting oncogenic miRs by using miRNA antagonists and replacement of tumor suppressor miRs to restore a loss-of-function [54].

### 6.1. miR-200 Family

Members of the miR-200 family (miR-141, miR-200a, miR-200b, miR-200c, and miR-429) are downregulated in the majority of ovarian cancers, as previously described [21, 23]. Marchini et al. reported that low levels of miR-200c can predict poor survival and are a biomarker of relapse in stage I epithelial ovarian cancer [28]. The miR-200 family plays a critical role in the suppression of epithelial-to-mesenchymal transition (EMT) and tumor cell migration, invasion, and metastasis by directly targeting* ZEB1* (zinc finger E-box-binding homeobox 1) and* ZEB2* [55, 56]. Both miR-141 and miR-200a target p38 and modulate the oxidative stress response, affecting tumorigenesis and chemosensitivity [55]. miR-200a or miR-200c inhibits cancer stem-like cell populations [56, 57]. Pecot et al. demonstrated that miR-200 members inhibit angiogenesis through direct and indirect mechanisms by targeting interleukin-8 and* CXCL1* secreted from the tumor epithelial and cancer cells. They showed the therapeutic potential of miR-200 delivery in treating ovarian cancer or other malignancies [58].

Furthermore, it has been reported that miR-200 family members are associated with chemosensitivity in ovarian cancer. Cochrane et al. found that class III tubulin (*TUBB3*), which encodes a tubulin isotype normally found only in neuronal cells, is a direct target of miR-200c [59, 60]. The restoration of miR-200c increased sensitivity to microtubule-binding chemotherapeutic drugs, paclitaxel, epothilone B, and vincristine and suppressed the expression of* TUBB3*. Van Jaarsveld et al. compared the miR expression profiles of cisplatin-sensitive and -resistant ovarian cancer cells, revealing that high expression of miR-141, miR-200c, miR-215, and miR-421 and low expression of miR-492-5p correlated with increased cisplatin resistance [61]. They also demonstrated that miR-141 directly targets* KEAP1*, and downregulation of* KEAP1* by miR-141 overexpression induced cisplatin resistance.

### 6.2. miR-199/214 Cluster

Chen et al. discovered that miR-199a regulates IKK expression, which modulates the inflammatory microenvironment in ovarian cancer [62]. Yin et al. showed that TWIST1 regulated the IKK/NF-*κ*B and PTEN/AKT pathways through the miR-199a-2/miR-214 cluster [63]. miR-199a also targets* CD44* to suppress the tumorigenicity and multidrug resistance of ovarian cancer-initiating cells [64]. Epigenetic silencing of miR-199b-5p is associated with chemoresistance in ovarian cancer through the activation of JAG1/Notch1 signaling [65]. Yang et al. showed that miR-214 induced cell survival and cisplatin resistance through direct targeting of* PTEN* and inactivation of the AKT pathway [66]. Joshi et al. found that the expression of miR-199a is reduced in cancer cells by hypoxic stimuli, and exogenous expression of miR-199a decreased cell migration and metastasis of ovarian cancer cells by targeting the 3′-UTRs of HIF-1*α* and HIF-2*α* [67].

### 6.3. let-7 Paralogs

The let-7 family includes 12 human homologs that are considered tumor suppressors. These miRs are located in cancer-associated regions or in fragile sites [68]. Johnson et al. reported that the let-7 family negatively regulates let-60/*RAS*, whose 3′-UTR contains multiple let-7 complementary sites [10]. In ovarian cancer, let-7 is downregulated. It also targets the embryonic gene high mobility group A2 (*HMGA2*) more efficiently than* RAS* during early cancer progression [69, 70]. Shell et al. demonstrated that let-7 and* HMGA2* can be predictors of prognosis and that loss of let-7 expression indicates less differentiated cancer [70]. High-grade serous ovarian carcinoma (HG-SOC) is a heterogeneous, poorly classified, and lethal disease. Recently, meta-analysis of its transcriptome revealed let-7b as an unfavorable prognostic biomarker that can predict molecular and clinical subclasses of HG-SOC [71]. A let-7b-defined 36-gene prognostic survival signature outperformed many clinicopathological parameters. As for let-7e, Cai et al. suggested that it might act as a promising therapeutic target for improving sensitivity to cisplatin in ovarian cancer [72].

### 6.4. miR-506

From integrated genomic analysis, 8 key miRs (miR-25, miR-29c, miR-101, miR-128, miR-141, miR-182, miR-200a, and miR-506) were predicted to regulate 89% of the miR targets in the network [26]. In follow-up functional experiments, overexpression of miR-506 in ovarian cancer cells augmented E-cadherin expression, inhibited cell migration and invasion, and prevented TGF-*β*-induced EMT by targeting* SNAI2*, a transcriptional repressor of E-cadherin. In an orthotopic ovarian cancer mouse model, nanoparticle delivery of miR-506 significantly reduced tumor growth. Liu et al. reported that miR-506 also suppressed ovarian cancer cell proliferation and induced senescence by directly targeting the CDK4/6-FOXM1 axis [73].

### 6.5. miR-92a

miR-92a is in the miR-17/92 family cluster, which includes miR-17, miR-18, miR-19a, mir-19b, miR-20, and miR-92. Ohyagi-Hara et al. described the involvement of miR-92a in the expression of integrin *α*5, a known key player in ovarian cancer adhesion and dissemination [74, 75]. The levels of integrin *α*5 and miR-92a expression were significantly and inversely correlated in ovarian cancer cells. The forced expression of miR-92a in cancer cells markedly suppressed peritoneal dissemination* in vivo*, suggesting that targeting miR-92a may prove to be a novel and effective gene therapy for patients with ovarian cancer.

### 6.6. miR-31

Mitamura et al. analyzed miR-associated paclitaxel (PTX) chemoresistance in ovarian cancer cells [76]. Lower expression of miR-31 and higher expression of MET (also known as c-Met or hepatocyte growth factor receptor) were significantly correlated with PTX resistance and poor prognosis in ovarian cancer patients. miR-31 directly targets the 3′-UTR of MET and increases the PTX sensitivity of ovarian cancer cells in an animal model. Creighton et al. comprehensively profiled the expression of miRs and mRNAs in serous ovarian cancers, cell lines, and normal ovarian epithelium [77]. They discovered that miR-31, the least regulated miR in serous ovarian cancer, repressed the cell cycle regulator* E2F2*, inhibited proliferation, and induced apoptosis. They revealed that loss of miR-31 is associated with defects in the TP53 (also called p53) pathway and functions in serous ovarian cancer, suggesting that patients with cancers that are deficient in TP53 activity might benefit from therapeutic delivery of miR-31.

### 6.7. miR-484

Vecchione et al. analyzed miR signatures associated with chemoresistance in 198 serous ovarian cancer samples along with clinical data and concluded that the presence miR-217, miR-484, and miR-617 was able to predict the chemoresistance of these tumors [78]. The response to chemotherapy is associated with tumor angiogenesis, and miR-484 has a potential to improve chemosensitivity through the modulation of tumor angiogenesis, by directly targeting* VEGFB* and* KDR* (formerly called* VEGFR2*).

### 6.8. Therapeutic Synergy between miR-520d-3p and EPHA2 siRNA

Nishimura et al. identified miR-520d-3p as a tumor suppressor upstream of* EPHA2*, whose expression correlated with favorable outcomes in clinical cohorts [79]. Dual inhibition of* EPHA2*, using* EPHA2* siRNA and nanoliposomes loaded with miR-520d-3p, showed better antitumor efficacy than either monotherapy* in vivo*. These data emphasize the feasibility of combined miRNA-siRNA therapy for cancer or other diseases.

## 7. miR and Tumor Microenvironment

The interaction of cancer cells with their microenvironment is essential for tumor development, tumor progression, and metastasis [80]. Tumor microenvironment is a collective term that includes the tumor's surrounding supportive stroma, the host immune system, and other humoral factors. Various miRs have the therapeutic potential by targeting not only tumor cells directly, but also cells surrounding tumor microenvironment.

### 7.1. Angiogenesis and miRs

Pecot et al. demonstrated that miR-200 inhibits angiogenesis through direct and indirect mechanisms by targeting interleukin-8 and CXCL1 that is secreted by tumor endothelial cells [58]. Using several experimental models, including models of ovarian cancer, they showed that the delivery of the members of the miR-200 family into the tumor endothelium led to marked reduction in metastasis and angiogenesis and induced vascular normalization, resulting in ovarian cancer regression. Xu et al. found that miR-145 acts as a tumor suppressor by indirectly downregulating the expression of hypoxia-inducible factor 1 (HIF-1) and vascular endothelial growth factor (VEGF) by targeting p70S6K1, in turn resulting in the inhibition of tumor growth and angiogenesis [81]. Similarly, miR-125b and miR-199a were also shown to act as tumor suppressors by targeting HIF-1*α* and VEGF in ovarian cancer cells, consequently reducing angiogenesis [82]. Lai et al. reported that miR-27a may play a central role in follicle-stimulating hormone- (FSH-) mediated angiogenesis in ovarian cancer. They showed that the ablation of miR-27a repressed FSH-induced expression of VEGF, Cox2, and survivin [83]. Because antiangiogenic therapy in ovarian cancer has been shown to be effective in several large phase III trials, these miRs could be used in the development of ovarian cancer therapies in the future [84, 85].

### 7.2. Cancer Associated Fibroblasts (CAFs) and miRs

Cancer cells change the surrounding normal stroma into tumor supportive environments during the processes of invasion and metastasis [86]. Cancer associated fibroblasts (CAFs) are a major component of the tumor stroma. They promote cancer cell invasion and enhance the viability of tumor cells. Mitra et al. found that, in ovarian CAFs, miR-31 and miR-214 are downregulated while miR-155 is upregulated compared to normal or tumor-adjacent fibroblasts [87]. Their results indicate that ovarian cancer cells reprogram fibroblasts to become CAFs through miRs and that targeting miRs in stromal cells has therapeutic potential.

### 7.3. Cancer-Associated Dendritic Cells (DCs) and miRs

Cancer-associated dendritic cells (DCs) represent the most frequent leukocyte subset to infiltrate solid tumors. These cancer-associated DCs are located around perivascular areas, where they deliver multiple proangiogenic and immunosuppressive mediators [88]. Huarte et al. demonstrated that the elimination of cancer-associated DCs delays ovarian cancer progression by boosting antitumor immunity [89]. Cubillos-Ruiz et al. showed that the activity of mature miR-155 can be augmented in tumor-associated DCs by delivering novel Dicer substrate RNA duplexes that mimic the structure of the endogenous precursor miR-155 hairpin [90]. The replenishing of miR-155 levels in DCs reprogrammed their immunosuppressive phenotype and boosted potent antitumor immune responses that abrogated the progression of established ovarian cancers.

## 8. Current Challenges in miRNA Delivery

The data presented in this review support a clinical role of miRs in ovarian cancer and suggest that miR-regulated pathways may be relevant targets in novel therapeutics. However, there remains a major challenge of miR-based cancer therapy with respect to systemic delivery* in vivo*. In particular, the problems related to specificity, efficiency, and safety pose major limitations. The keys for miR drug development are that the chemical structure must be stable* in vivo* and cell-permeable and should hybridize to the miR of interest with high specificity and affinity. Techniques for chemical modifications have been applied to enhance oligonucleotide stability and to acquire increased resistance to nucleases. Examples include 2-O-methyl-group- (OMe-) modified oligonucleotides and locked nucleic acid- (LNA-) modified oligonucleotides [91]. LNA-antimir-122 (miravirsen) is the first drug to successfully enter phase II trials for the treatment of hepatitis C virus (HCV) infection [92]. Thirty-six patients were randomly assigned to receive five weekly subcutaneous injections of miravirsen at doses of 3, 5, or 7 mg/kg or placebo over a 29-day period. Miravirsen resulted in a dose-dependent reduction in HCV RNA levels, and there were no observed dose-limiting adverse effects or escape mutations in the miR-122 binding sites of the HCV genome. Systemic delivery of miRNA, like that of other types of nucleic acids, activates the innate immune system leading to unexpected toxicities and significant undesirable side effects. When anti-inflammatory miRs are concurrently delivered as therapeutic agents, they may suppress the systemic immune response instead of causing immune toxicity [41]. Moreover, one of the biggest issues regarding miR therapy is the off-target effect of miRNAs. Since miRs are designed to target multiple pathways via imperfect matching with 3′-UTRs, they may cause unwanted silencing of tumor suppressor genes. Such off-target gene silencing may cause potential toxicities and reduced therapeutic effects [41]. A multifunctional nanoparticle delivering miRs, siRNAs, or miRNA cocktails to silence several oncogenic pathways and activate tumor suppressive ones may minimize unintended off-target effects and maximize the therapeutic effect. Although these problems remain unsolved, conquering them may render miR-based therapy an important armament for cancer therapy in the future. Further studies are required in order to successfully apply therapeutic miRs to ovarian cancer, and this could realize a potential use of miRs to drastically improve the prognosis of this disease.

## 9. Conclusion

Since 2005 and the discovery of miR-15a and miR-16-1 deletions in B-CLL [12], there have been an enormous number of reports regarding miR dysregulation in various cancer types. In addition to transcriptional regulation, posttranscriptional repression by miRs contributes to every cell/tissue function by fine-tuning large networks of genes. In the ovarian cancer field, many miR signatures from tumor cells/tissues or serum/plasma have been described so far. Since early detection tools are lacking, ovarian cancer is often diagnosed at a late stage. This substantially contributes to the high mortality rate of ovarian cancer. This review summarized that miR expression profiles are quite different in ovarian cancer compared to normal control tissue. Thus, in the near future, screening serum/plasma miRs might contribute to improved prognosis of ovarian cancer by enabling diagnosis at an early stage noninvasively.

**Figure 1:**
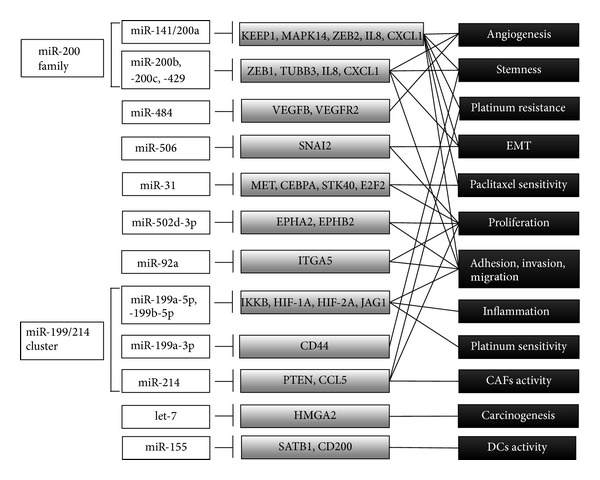
Schema of mechanism and target genes of potential therapeutic miRs for ovarian cancer.

Emerging evidence strongly supports the rationale that inhibition of overexpressed oncogenic miRs or substitution of tumor suppressive miRs might be novel treatment strategies for ovarian cancer therapy, as summarized in Table 3 and Figure 1. Optimization of the stabilizing method for miRs, improvement in delivery methods, and the control of off-target effects induced by miRs delivery appear to be the keys to future clinical applications.

## Figures and Tables

**Table 1:** Potential prognostic miRs for ovarian cancer, which are significant in multivariate analysis (modified from [27]).

Reference	microRNA	Patients	Prognosis	Endpoint
[29], 2013	miR-21 (Serum)	EOC	Poor	OS
[30], 2014	miR-25	EOC	Poor	OS
[31], 2009	miR-29b	SAC	Poor	DFS
[32], 2012	miR-100	EOC	Good	OS
[33], 2014	miR-150	EOC	Good	PFS, OS
[34], 2012	miR-187	EOC	Good	RFS, OS
[35], 2009	miR-200a	EOC	Good	RFS, OS
[28], 2011	miR-200c	EOC (Stage 1)	Good	PFS, OS
[36], 2013	miR-203	EOC	Poor	PFS, OS
[37], 2013	miR-221	EOC	Poor	OS
[38], 2010	miR-221/222 ratio	EOC	Good	OS
[39], 2014	miR-335	EOC	Good	RFS, OS
[40], 2011	miR-410 and miR-645	EOC	Good	OS

EOC: epithelial ovarian cancer; SAC: serous adenocarcinoma; OS: overall survival; DFS: disease-free survival; PFS: progression-free survival; RFS: recurrent-free survival.

**Table 2:** Potential diagnostic miRs for ovarian cancer.

Reference	Sample	Elevated miR	Decreased miR	Tumor histology (*n*)	Control (*n*)
[47], 2008	Exosome (serum)	miR-21, miR-141, miR-200a, miR-200c, miR-200b, miR-203, miR-205, miR-214		SAC (50)	BOA (10), HC (10)
[48], 2009	Serum	miR-21, miR-92, miR-93, miR-126, miR-29a	miR-155, miR-127, miR-99b	SAC (17), CAC (6), EAC or MAC (5)	HC (15)
[49], 2010	Whole blood	miR-30c-1-3p	miR-181a-3p, miR-342-3p, miR-450-5p	SAC (21), EAC (2), other (1) (relapsed)	HC (15)
[50], 2012	Serum	miR-200a, miR-200b, miR-200c		SAC (28)	HC (28)
[51], 2013	Serum		miR-132, miR-26a, let-7b, miR-145	SAC (18)	HC (12)
[52], 2013	Plasma	miR-16, miR-21, miR-191 (CAC, EAC)		SAC (21), CAC (7), EAC (6), other (1)	EM (33), HC (20)
		miR-16, miR-191, miR-4284 (SAC)			
[53], 2013	Plasma	miR-205	let-7f	SAC (179), CAC (15), EAC (86), MAC (33), other (47)	HC (200)

SAC: serous adenocarcinoma; CAC: clear cell adenocarcinoma; EAC: endometrioid adenocarcinoma; MAC: mucinous adenocarcinoma; BOA: benign ovarian adenoma; HC: healthy control; EM: endometriosis.

**Table 3:** Potential therapeutic miRs for ovarian cancer.

miRNA		Target gene	Cellular function	Ovarian cancer cell line	Reference
miR-200 family	miR-141	KEEP1	Cisplatin resistance	A2780 (EOC), TOV112D (EAC), TOV21G (CAC)	[61]
	miR-141, miR-200a	MAPK14	Oxidative stress response, pactitaxel sensitivity	SKOV3 (EOC)	[55]
	miR-200a	ZEB2	EMT, stemness, migration, invasion	OVCAR3 (EOC)	[56]
	miR-200c	ZEB1, TUBB3	EMT, stemness, adhesion, migration, invasion, paclitaxel sensitivity	HEY (SAC), SKOV3 (EOC)	[57, 59, 60]
	miR-200a, miR-200b	IL8, CXCL1	Angiogenesis	HeyA8 (SAC), ES2 (CAC)	[58]

miR-199/214 cluster	miR-199a-5p	IKKB, HIF-1A, HIF-2A	Inflammation, chemosensitivity, migration, metastasis	A2780 (EOC), R454 (EOC), 01-28 (EOC), R182 (EOC), 01-19B (EOC), R1185 (EOC), primary culture	[62, 63, 67]
	miR-199a-3p	CD44	Stemness, chemosensitivity	primary culture	[64]
	miR-199b-5p	JAG1	Cisplatin sensitivity	A2780 (EOC), OV119 (EOC)	[65]
	miR-214	PTEN, CCL5	Proliferation, cell survival, cisplatin resistance, CAFs activity	A2780 (EOC), HeyA8 (SAC), SKOV3ip1 (EOC), OV119 (EOC), primary culture	[63, 66, 87]

let-7		HMGA2	Carcinogenesis	A2780 (EOC), HeyA8 (SAC), IGROV-1 (EOC)	[69, 70]

miR-506		SNAI2	EMT	HeyA8 (SAC), SKOV3 (EOC), OVCA420 (EOC), OVCA433 (EOC)	[26]
		CDK4, CDK6	Proliferation, senescence	HeyA8 (SAC), SKOV3 (EOC), OVCA432 (EOC), OVCA433 (EOC)	[73]

miR-92a		ITGA5	Adhesion, invasion, proliferation	A2780 (EOC), SKOV3ip1 (EOC), OVISE (CAC)	[74]

miR-31		MET	Paclitaxel sensitivity	KFr13 (EOC)	[76]
		CEBPA, STK40, E2F2	Proliferation	SKOV3 (EOC), OVCAR8 (EOC), OVCA433 (EOC)	[77]

miR-484		VEGFB, VEGFR2	Angiogenesis	SKOV3 (EOC), MDAH-2274 (EOC)	[78]

miR-502d-3p		EPHA2, EPHB2	Proliferation, migration, invasion	HeyA8 (SAC), SKOV3ip1 (EOC), ES2 (CAC)	[79]

miR-155		SATB1, CD200	DCs activity	ID8 (EOC, mouse)	[90]

CAFs: cancer associated fibroblasts; EMT: epithelial-mesenchymal transition; DCs: dendritic cells; EOC: epithelial ovarian cancer; EAC: endometrioid adenocarcinoma; CAC: clear cell adenocarcinoma; SAC: serous adenocarcinoma.
